# Melatonin Supplementation during the Late Gestational Stage Enhances Reproductive Performance of Sows by Regulating Fluid Shear Stress and Improving Placental Antioxidant Capacity

**DOI:** 10.3390/antiox12030688

**Published:** 2023-03-10

**Authors:** Likai Wang, Laiqing Yan, Qi Han, Guangdong Li, Hao Wu, Xiao Ma, Mengmeng Zhao, Wenkui Ma, Pengyun Ji, Ran Zhang, Guoshi Liu

**Affiliations:** 1 State Key Laboratory for Agrobiotechnology, College of Biological Sciences, China Agricultural University, Beijing 100193, China; 2 National Engineering Laboratory for Animal Breeding, Key Laboratory of Animal Genetics and Breeding of the Ministry of Agricultural, Beijing Key Laboratory for Animal Genetic Improvement, College of Animal Science and Technology, China Agricultural University, Beijing 100193, China

**Keywords:** melatonin, swine reproduction, placenta, oxidative stress, fluid shear stress, Nrf2

## Abstract

In this study, the effects of daily melatonin supplementation (2 mg/kg) at the late gestational stage on the reproductive performance of the sows have been investigated. This treatment potentially increased the litter size and birth survival rate and significantly increased the birth weight as well as the weaning weight and survival rate of piglets compared to the controls. The mechanistic studies have found that these beneficial effects of melatonin are not mediated by the alterations of reproductive hormones of estrogen and progesterone, nor did the glucose and lipid metabolisms, but they were the results of the reduced oxidative stress in placenta associated with melatonin supplementation. Indeed, the melatonergic system, including mRNAs and proteins of AANAT, MTNR1A and MTNR1B, has been identified in the placenta of the sows. The RNA sequencing of placental tissue and KEGG analysis showed that melatonin activated the placental tissue fluid shear stress pathway to stimulate the Nrf2 signaling pathway, which upregulated its several downstream antioxidant genes, including MGST1, GSTM3 and GSTA4, therefore, suppressing the placental oxidative stress. All these actions may be mediated by the melatonin receptor of MTNR1B.

## 1. Introduction

During the process of pregnancy, the placenta (a temporary organ) is formed between the mother and the fetus. The placenta provides an interface between the mother and the fetal circulation for dual directional transportations of nutrients, oxygen and metabolic wastes, which is essential for proper fetal growth [1]. Therefore, the abnormal placental function will lead to a variety of pregnancy disorders, including intrauterine growth restriction, pre-eclampsia, and abortion. For example, the placental malfunction associated abnormal intrauterine environment during the embryonic period not only inhibits the fetus’ development and growth but also increases the risk of diabetes, hypertension and cardiovascular diseases after birth [2]. Even though multiple risk factors are attributed to placental dysfunctions, one of them is oxidative stress [3,4]. It has been reported that maternal exposure to high levels of reactive oxygen species (ROS) during pregnancy impairs placental function and leads to poor outcomes in pregnancy [5].

Oxidative stress can cause cell damage and, if not treated properly, ultimately will result in cell death [6]. Heat or cold stress, obesity, viral infection and other insults during pregnancy will increase the level of placental oxidative stress and jeopardize placental function with abnormal fetal development [7,8,9,10]. Specific to sows, the late gestation period is a critical stage for the development and growth of piglets. At this stage, the placenta needs to support the rapid growth of the fetus; therefore, it consumes a lot of energy, which will increase the maternal metabolic burden and oxidative stress [11]. To retard this placental oxidative stress, antioxidants have been used as effective strategies to prevent the adverse outcomes of pregnancy in animals [5].

Melatonin (*N*-acetyl-5-methoxytryptamine) is an antioxidant mentioned above. Melatonin is an indolamine molecule present in bacteria found in mammals [12]. It exhibits a spectrum of biological functions, including regulation of biological rhythm, sleep, immune function and reproductive physiology [13]. Importantly, melatonin and its metabolites have potent anti-oxidative activity, which not only directly scavenges free radicals but also upregulates the expression of antioxidant enzymes [14,15]. Evidence has shown that melatonin can be synthesized in the human placenta locally. This is indicated by the melatonin production positively relates to the function of the placenta. For example, the level of melatonin in peripheral blood gradually increases with the progress of human pregnancy, reaches a peak in the prenatal period, and returns to a normal level after delivery [16]. In addition, melatonin receptors 1A (MTNR1A) and 1B (MTNR1B) have also been expressed in human placental tissues [17]. Treatment of cytotrophoblast cells with melatonin improves cellular syncytization and promotes the synthesis and secretion of human chorionic gonadotophin (hCG) [17]. Under placental dysfunction with fetal growth restriction, the expressions of MTNR1A and MTNR1B are downregulated, and the level of melatonin in peripheral blood is significantly decreased, suggesting an important role of melatonin in the maintenance of human placental function [18,19]. Melatonin supplementation in rats between days 15 and 20 of gestation improves their placental function and puppy’s birth weight via upregulating the expression level of antioxidant enzymes in placental tissue under the condition of malnutrition [20]. Renshall et al., have reported that in mice with normal pregnancy, melatonin supplementation between days 12.5 and 18.5 of the gestation stage also increases fetal birth weight [21]. Recently, Peng et al., supplemented melatonin to sows from the period of fertilization to parturition [22]. They observed that the proportion of piglets with birth weight <900 g was reduced, but the average birth weight of piglets exhibited no significant difference compared to the control. They attributed these to the antioxidant properties of melatonin. The early intervention of pregnant animals with antioxidants is debatable since an appropriate ROS level is essential for placental angiogenesis at the initial stage of gestation. For example, Yang et al., found that the antioxidant treatment can significantly reduce the blood vessel density of the placenta in the mouse placenta formation stage and also significantly reduce fetal birth weight and aggravate the intrauterine growth restriction phenotype [23,24]. Specific to sows, the late gestation stage is the most important period of fetal growth and development, which will produce a large number of ROS and easily lead to intrauterine growth restriction [25]. 

Therefore, in the current study, melatonin was given to the sows at their gestation days between 90–114 to investigate whether melatonin supplementation at the late gestation stage could improve placental function and thus improve fetal growth. If it does, what are the underlying molecular mechanisms and signal transduction pathways? The results will provide valuable information for the application of melatonin to improve reproductive efficiency in swine or even in other animals. 

## 2. Materials and Methods

### 2.1. Chemicals and Agents

Melatonin was purchased from Sigma Company (St. Louis, MO, USA). The glucose detection kit (F006-1-1), triglyceride detection kit (A110-1-1) and malondialdehyde detection kit (A003-1-2) were purchased from Nanjing Jiancheng Biological Engineering Research Institute Co., Ltd. (Nanjing, China) The prolactin detection kit (P07PZB), progesterone detection kit (P08PZB), estradiol detection kit (B05PZB), and cortisol detection kit (D10PZB) were purchased from Beijing North Biotechnology Research Institute Co., Ltd. Antibodies against AANAT (ab3505), MTNR1A (ab203038) and MTNR1B (ab203346) were purchased from Abcam (Cambridge, MA). The secondary antibody conjugated with CoraLite594 (SA00013-4) was purchased from Proteintech (Wuhan, China). The secondary antibody conjugated with Peroxidase (ZB-2301) was purchased from Beijing Zhongshan Jinqiao Biotechnology Co., Ltd. (Beijing, China).

### 2.2. Animals

The design of animal experiments in this study complies with the regulations of the Animal Welfare Committee of China Agricultural University (permission number: AW60103202-3-1). The sows (Large White × Landrace) are from Yantai Fuzu Food Co., Ltd. in Shandong province. All sows were first farrowing sows (approximately one year old) with similar backfat thickness.

### 2.3. Animal Study Design

Twelve sows with similar body weight (around 200 kg) at 90 days of gestation were divided into a control group and a melatonin group, with 6 sows in each group. The environmental light/dark cycle was 12/12 h, and the temperature was controlled at 20–25 °C. These sows were given the same basal diet twice a day, at 7:00 am and 5:00 pm, but the experimental group was given additional melatonin. Melatonin was given daily at 7:00 am at a dose of 2 mg/kg added directly to the feed to ensure complete consumption. At 107 days of gestation, the sows were transferred to the farrowing room. The treatment was continued from 90 days of gestation to parturition.

### 2.4. Blood Sample Collection

In order to avoid premature labor caused by the stress of frequent blood collection, we decided to reduce the blood collection as less as possible. Therefore, we selected to collect blood every 10 days after melatonin treatment (corresponding to the middle and late stages of the melatonin feeding cycle), i.e., on days 100 and 112 of gestation, respectively. Blood was collected from the antecubital vein with 8 mL volume each time, and blood samples were placed in 10 mL sterile heparinized vacuum tubes and immediately centrifuged at 3500 g for 15 min. The plasma was separated and stored at −80 °C for further analysis of melatonin, hormone and biochemical indexes.

### 2.5. Measurement of Piglet Weight after Birth and Placental Tissue Collection

After birth, the total number of piglets was recorded, including the survived and dead (One sow in the control group had premature birth and was not included in the statistics). The weight of the piglets was measured after the piglet coat was dried. The expelled placental tissue was collected, rinsed in PBS and immediately placed in liquid nitrogen for rapid freezing (about 5 g per placenta, 3 to 4 cm from the point of umbilical cord insertion). A 2 mL of colostrum sample was collected from each sow and stored at −80 °C for future analysis.

### 2.6. Melatonin Detection

The serum or colostrum was mixed with methanol at a ratio of 1:4, vortex-oscillated, and centrifugated (4 °C, 12,000 rpm) to collect supernatant, filtered with 0.22 µm of filter, and the effluent was used for melatonin detection. Melatonin detection was carried out in the central laboratory of the Beijing Institute of Animal Science, Chinese Academy of Agricultural Sciences, by the high-performance liquid mass spectrometer (Agilent1290-G6470, Santa Clara, CA, USA).

### 2.7. Detection of Hormone and Biochemical Indicators

Serum reproductive hormones, including progesterone, estrogen, cortisol and prolactin, were determined by radioimmunoassay (Xi’an Nuclear Instrument Factory, XH6080). For progesterone and cortisol detection, 50 μL serum samples, 100 μL ^125^I-progesterone or 100 μL ^125^I-cortisol, then 100 μL rabbit anti-progesterone antibody or 100 μL rabbit anti-cortisol antibody, were pooled together, respectively. The samples were incubated at 37 °C for 1 h, then 500 μL of immunoseparation agent was added and placed at room temperature for 15 min. After that, the samples were centrifuged at 3500 rpm/15 min to remove the supernatant, and the radioactive count of the pallets was detected. For prolactin detection, 100 μL serum, 100 μL of ^125^I-prolactin and 100 μL of rabbit anti-prolactin antibody were mixed and incubated at 4 °C for 24 h, then 500 μL of immunoseparation agent were added and placed the samples at room temperature for 15 min, after that, the samples were centrifuged at 3500 rpm/15 min to remove the supernatant, and the radioactive count of the pallets was detected. For estradiol detection, 100 μL serum, add 100 μL of ^125^I-estradiol and 100 μL of rabbit anti-estradiol antibody were pooled together and incubated at 37 °C for 1 h, then 500 μL of immunoseparation agent were added, and the samples were placed at room temperature for 15 min, after that, the samples were centrifuged at 3500 rpm/15 min to remove the supernatant, and the radioactive count of the pallets was detected. The serum glucose was measured by use of the glucose oxidase method following the manufacturer’s instructions, and the absorption at the wavelength of 505 nm was detected to calculate the glucose content (Glucose concentration = sample absorption value/calibrator absorption value × calibrator concentration) [7]. The serum triglyceride was measured by use of the enzyme method following the manufacturer’s instructions, and the absorption at the wavelength of 546 nm was detected to calculate the triglyceride (Triglyceride concentration = sample absorption value/calibrator absorption value × calibrator concentration) [7]. The serum malondialdehyde was measured by use of the thiobarbituric acid method following the manufacturer’s instructions, and the absorption at the wavelength of 532 nm value was detected to calculate the malondialdehyde content ((Sample absorbance value − blank absorbance value)/(standard absorbance value − blank absorbance value) × Standard concentration) [7]. 

### 2.8. Real-Time Quantitative PCR

Total RNA was extracted from fresh placental tissue using TRIzol reagent (Invitrogen, Carlsbad, CA, USA). 1 μg total RNA was synthesized using PrimeScript™ RT Master Mix (TaKaRa, Kusatsu, Japan). Primers selected for PCR analyses were designed using Primer5 and are listed in Supplementary Table S1. The total reaction volume (20 μL) comprised 2 μL of cDNA template solution, 10 μL of SYBR Green PCR Master Mix (Roche, Basel, Switzerland), 6.4 μL of water, and 0.8 μL of each primer. The RT PCR program included a 10 min incubation at 95 °C, followed by 40 cycles of denaturation for 15 s at 95 °C and annealing and extension for 30 s at 60 °C. The mRNA expression levels of the target genes were normalized to the expression of GAPDH. Relative gene expression was expressed as a ratio of the target gene to the control gene using the formula 2^−(ΔΔCT)^.

### 2.9. Western Blotting

Total cell lysates were prepared from fresh placental tissue using a cell lysis buffer RIPA (Beyotime, Shanghai, China) containing 1% PMSF. The placental tissue was homogenatized by a Vortex generator (Roche, Basel, Switzerland, USA), and the supernatant was collected after centrifugation. The protein concentrations were determined using an Enhanced BCA Protein Assay Kit (Beyotime, Shanghai, China). The same volume of 5 × loading buffer was mixed with the samples, which were subject to boiling for 10 min. After separation on 10% SDS-PAGE, the samples were transferred to a 0.45 mm PVDF membrane followed by a 2-h blocking with 5% non-fat dry milk at room temperature. Then the membranes were then incubated overnight at 4 °C with respective primary antibodies (dilution rate 1:1000). After incubation with a secondary antibody (dilution rate 1:10,000) for another 1 h, then the protein expression was detected by a Super Signal™ West Pico PLUS Chemiluminescent Substrate (Thermo, Waltham, MA, USA). 

### 2.10. Immunofluorescence Detection of AANAT, MTNR1A and MTNR1B

Placental tissues fixed in 4% paraformaldehyde were paraffin-embedded and sectioned at 5 μm thickness for AANAT, MTNR1A and MTNR1B immunofluorescence. The placental tissue sections were dewaxed and rehydrated; sodium citrate was used for thermal repair and then incubated with a catalase blocker for 10 min. After rinsed with PBS, a monoclonal antibody of AANAT, MTNR1A and MTNR1B were added for overnight incubation (dilution rate 1:100). A secondary antibody coupled with CoraLite594 (dilution rate 1:100) was incubated and nucleated with 4′,6-diaminyl-2-phenylindole (DAPI) for observation. Finally, the slides were visualized under a fluorescent microscope (Nikon Eclipse C1, Tokyo, Japan).

### 2.11. RNA-Seq for Placenta Tissue

Total RNA was extracted from placenta tissue by TRIzol (Ambion, 15596026), and genomic DNA was removed by DNase I (TaKara). The quality of RNA samples was detected by 2100 Bioanalyser (Agilent, Palo Alto, CA, USA) and ND-2000 (NanoDrop Technologies, Wilmington, DE, USA). After the samples were qualified (OD260/280 = 1.8–2.2, OD260/230 ≥ 2.0, RIN ≥ 6.5), 28S:18S ≥ 1.0, >2 mg) were sequenced. 

TruSeqTM RNA Sample Preparation Kit (Illumina, San Diego, CA, USA) was used for the construction of the RNA library. SuperScript double-stranded cDNA synthesis Kit (Invitrogen, Carlsbad, CA, USA) was used to inversely synthesize cDNA and form a stable double-stranded structure. After cDNA enrichment by PCR (sample Preparation Kit (Illumina, San Diego, CA, USA) Kit), DNA clean Beads (DNA Clean Beads) are screened for 200–300 bp bands. After quantified by TBS380 (Picogreen), Illumina HiSeq XTEN/NovaSeq 6000 sequencing platform was used for high-throughput sequencing with a read length of PE150. 

### 2.12. Statistical Analysis

Data are expressed as the means ± SEM. A two-tailed Student’s *t*-test was used for statistical analysis using GraphPad Prism Software 7.00. *p*-values < 0.05 were considered to be statistically significant.

## 3. Results

### 3.1. Effects of Melatonin Supplementation on Levels of Melatonin in Blood and Colostrum

**Figure 1:**
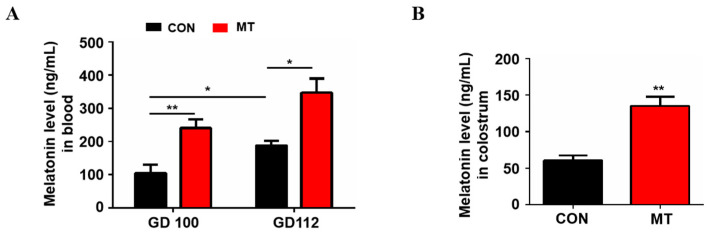
Melatonin level in blood and colostrum. (**A**) Blood melatonin levels at day 100 and 112 of gestation, respectively (*n* = 4 for per group). (**B**) Melatonin level in colostrum (*n* = 3 for per group). Data are presented as means ± SEM. * means *p* < 0.05. ** means *p* < 0.01. GD 100 = gestation day of 100, GD 112 = gestation day of 112, CON = control, MT = melatonin.

The blood melatonin levels at 100 and 112 days of gestation are shown in Figure 1A. Compared to the control group, melatonin supplementation significantly increased the blood melatonin levels in sows at day 100 and 112 of gestation, respectively (*p* < 0.05). The results also showed that blood melatonin concentrations in control sows at 112 days of gestation (188.1 ± 13.02 ng/mL) were significantly higher than those at 100 days of gestation (103.8 ± 26.49 ng/mL) (*p* < 0.05, Figure 1A). In addition, the melatonin level in the colostrum of sows was also significantly increased after melatonin feeding (61.0 ± 6.68 ng/mL versus 135.5 ± 12.20 ng/mL, *p* < 0.01, Figure 1B). 

### 3.2. Effects of Melatonin Supplementation on Reproductive Performance of Sows

**Figure 2:**
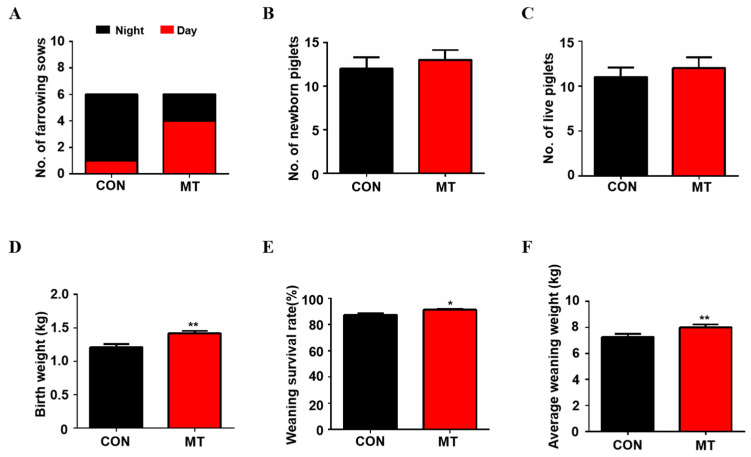
Reproductive Performance of sows with melatonin supplementation. (**A**) The number of sows giving birth during the day and night (*n* = 6). (**B**) The average number of newborn piglets (*n* = 5 for CON, *n* = 6 for MT group). (**C**) The average number of born alive (*n* = 5 for CON, *n* = 6 for MT group). (**D**) Average birth weight of piglets (*n* = 55 for CON, *n* = 72 for MT group). (**E**) Average weaning survival rate (*n* = 5 for CON, *n* = 6 for MT group). (**F**) Average weaning weight of piglets (*n* = 30 for CON, *n* = 36 for MT group), to avoid the stress of all piglets during weighing and affecting their growth, only six piglets from each sow were randomly selected for weighing). Data are presented as means ± SEM. * *p* < 0.05. ** *p* < 0.01. CON = control, MT = melatonin.

The results showed that the birth time of sows in the control group was mainly at night, while the birth time of sows in the melatonin supplementation group was mainly during the day (Figure 2A). As shown in Figure 2B,C, melatonin feeding exhibited the tendency to increase the average total litter size (12.0 ± 1.30 versus 13.0 ± 1.13) and average total live births of the piglets (11.0 ± 1.05 versus 12.4 ± 1.36) compared to the control group, but the difference did not reach to the statistical significance (*p* > 0.05). It is interesting to note that melatonin significantly increased piglet weight at birth compared to the control group (1.4 ± 0.04 kg versus 1.2 ± 0.05 kg, *p* < 0.01, Figure 2D) while no sows were found to have difficulty in farrowing due to the increased body weight of the litters in the melatonin group. The weaning survival rate (91.27 ± 0.81 versus 87.46 ± 1.34, *p* < 0.05, Figure 2E) and weight (8.2 ± 0.18 kg versus 7.2 ± 0.18 kg, *p* < 0.01, Figure 2F) of the piglets were also significantly higher in the melatonin-added group than in the control group at 21 days after birth. 

### 3.3. Effects of Melatonin Supplementation on Blood Reproductive Hormone and Other Biochemical Parameters

**Figure 3:**
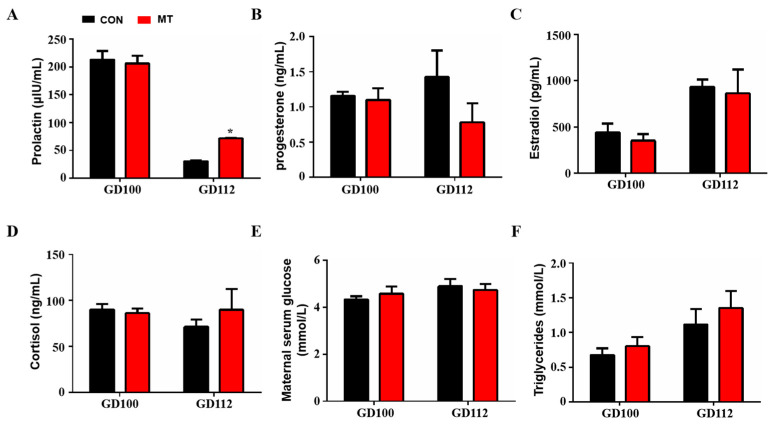
Effects of melatonin supplementation on blood hormone and other biochemical indices. (**A**) Prolactin concentration (GD100, *n* = 5, GD112, *n* = 3). (**B**) Progesterone concentration (GD100, *n* = 5, GD112, *n* = 3). (**C**) Estrogen concentration (GD100, *n* = 5, GD112, *n* = 3). (**D**) Cortisol concentration (GD100, *n* = 5, GD112, *n* = 3). (**E**) Glucose concentration (GD100, *n* = 5, GD112, *n* = 3). (**F**) Triglyceride concentration (GD100, *n* = 5, GD112, *n* = 3). Data are presented as means ± SEM. * *p* < 0.05. GD 100 = gestation day of 100, GD 112 = gestation day of 112, CON = control, MT = melatonin.

The results showed that melatonin supplementation did not change blood prolactin levels at 100 days of gestation but significantly increased prolactin levels at 112 days of gestation (GD 100: 213.2 ± 15.42 µIU/mL versus 206.2 ± 13.98 µIU/mL, GD 112: 30.3 ± 1.38 µIU/mL versus 71.78 ± 0.03 µIU/mL) (*p* < 0.05, Figure 3A). Melatonin supplementation in late pregnancy did not change the concentrations of progesterone, estradiol and cortisol in the blood (*p* > 0.05, Figure 3B–D). In addition, melatonin supplementation in late gestation did not affect glucose and triglyceride levels in the peripheral blood of sows (*p* > 0.05, Figure 3E,F).

### 3.4. Effects of Melatonin Supplementation on Melatonin Synthetic Enzyme AANAT and Melatonin Receptor Expression in Placental Tissue

**Figure 4:**
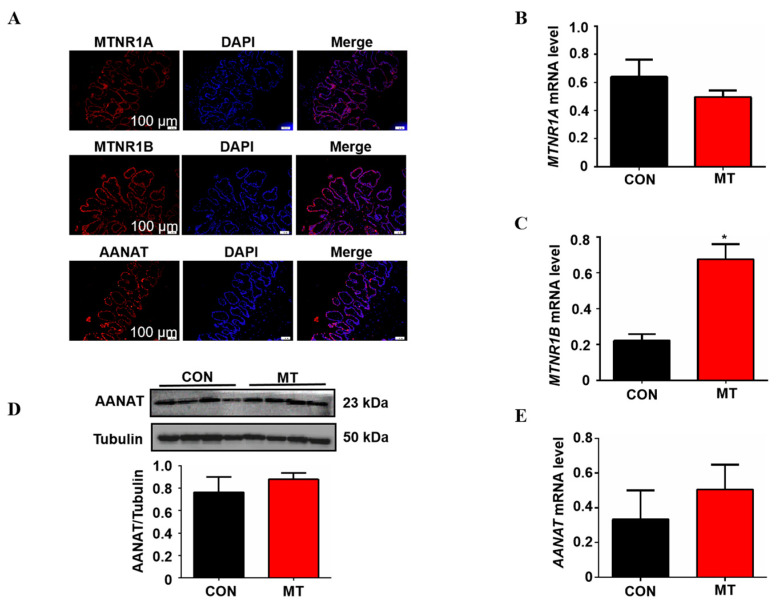
Detection of the melatonergic system in placental tissue of sows and effects of melatonin supplementation on it. (**A**) Location of MTNR1A, MTNR1B and AANAT in placental tissue. (**B**,**C**) The mRNA expression levels of MTNR1A and MTNR1B (*n* = 5). (**D**) The protein expression level of AANAT (*n* = 4). (**E**) The mRNA expression levels of AANAT (*n* = 5). Data are presented as means ± SEM. * *p* < 0.05. CON = control, MT = melatonin.

In this study, the expression of melatonin synthesizing rate-limiting enzyme AANAT and melatonin receptors MTNR1A and MTNR1B was detected by immunofluorescence in the placenta. The results showed that AANAT, MTNR1A and MTNR1B were expressed in the pig placenta and were mainly located in the outermost placental trophoblast cells (Figure 4A). Melatonin supplementation significantly increased the mRNA level of MTNR1B in placental tissue but did not affect the expression of MTNR1A compared with the control group (Figure 4B,C). In addition, Q-PCR and Western blot were used to detect the AANAT expression, as shown in Figure 4D,E; melatonin supplementation did not change the mRNA and protein level of AANAT in the placenta (*p* > 0.05). 

### 3.5. Effects of Melatonin Supplementation on the Transcriptome of Placental Tissue of Sows

**Figure 5:**
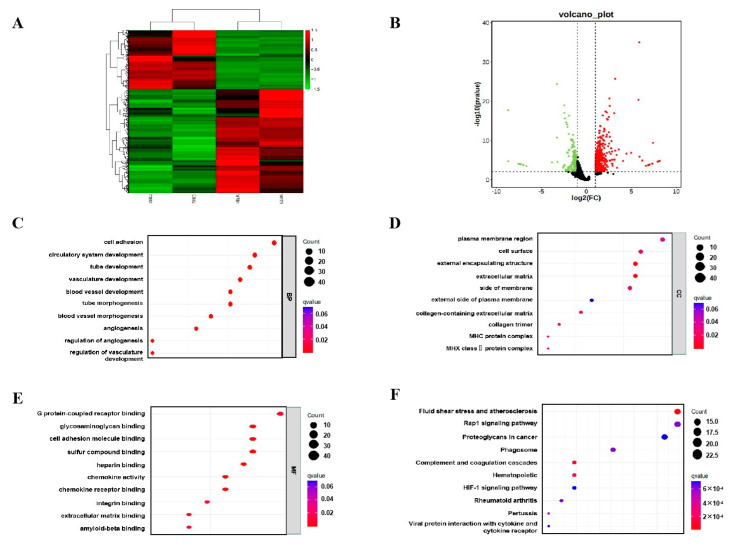
Effects of melatonin supplementation on the transcriptome of placental tissue. (**A**) Cluster analysis. (**B**) Expression difference analysis. (**C**) Analysis of biological processes in GO. (**D**) Analysis of cellular components in GO. (**E**) Analysis of molecular function in GO. (**F**) KEGG enrichment analysis.

Transcriptome sequencing was performed on the placental tissues. As shown in Figure 5A, there were significant differences in gene expression in the placenta between the melatonin supplementation group and the control group. A total of 758 differentially expressed genes (DEGs) were screened, including 484 upregulated and 274 down-regulated genes (Figure 5B). The biological features of DEGs were analyzed using Gene ontology (GO) analysis. The differentially expressed genes obtained from GO enrichment analysis were classified according to biological process (BP), cellular component (CC) and molecular function (MF). The most remarkably enriched BP terms were cell adhesion (GO:0007155), GC terms were plasma membrane region (GO:0098590), and MF terms were G protein-coupled receptor binding (GO:0001664) (Figure 5C–E). Kyoto Encyclopedia of Genes and Genomes (KEGG) is a comprehensive data library that combines information on genomic, chemical and system functions. The most significantly enriched pathways for DEGs were fluid shear stress and atherosclerosis, rap1 signaling pathway and proteoglycans in cancer (Figure 5F). Based on the results of further analysis, we suggest that Fluid shear stress and atherosclerosis signaling pathways may mediate the effects of melatonin on placental function.

### 3.6. Placental Tissue Antioxidant Capacity Test

**Figure 6:**
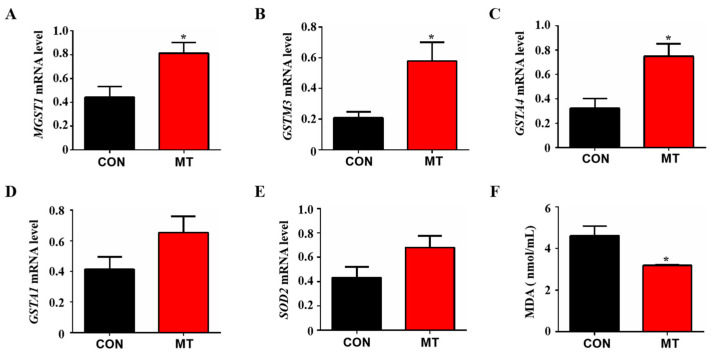
Effects of melatonin supplementation on the antioxidant capacity of placental tissue. (**A**) The mRNA expression levels of MGST1 (*n* = 5). (**B**) The mRNA expression levels of GSTM3 (*n* = 5). (**C**) The mRNA expression levels of GSTA4 (*n* = 5). (**D**) The mRNA expression levels of GSTA1 (*n* = 5). (**E**) The mRNA expression levels of SOD2 (*n* = 5). (**F**) MDA concentration in peripheral blood (*n* = 3). Data are presented as means ± SEM. * *p* < 0.05. CON = control, MT = melatonin.

Fluid shear stress and atherosclerosis are antioxidant-related pathways that can activate the Nrf2 signaling pathway to regulate the expression of many antioxidant genes. The results of RNA sequencing analysis showed that many antioxidant genes were enriched in Fluid shear stress and atherosclerosis pathway, including MGST1, GSTM3, GSTA1, SOD2 and GSTA4. Therefore, Q-PCR was performed to verify these differential genes. The results showed that melatonin supplementation significantly increased the expression levels of antioxidant genes MGST1, GSTM3 and GSTA4 in placental tissue compared to control (*p* < 0.05, Figure 6A–C), while no significant differences were observed as to the expression of GSTA1 and SOD2 between the groups (*p* > 0.05, Figure 6E). In addition, the level of malondialdehyde (MDA) in the peripheral blood of melatonin-treated sows was significantly lower than that in the control group (*p* < 0.05, Figure 6F). These results indicate that melatonin can improve the anti-oxidative stress ability of placental tissue by regulating fluid shear stress.

## 4. Discussion

As a “transport station”, the placenta plays an important role in transporting nutrients, oxygen/carbon dioxide and metabolic waste between mother and fetus, and thus, the placenta’s health is the premise of the normal growth and development of the fetus. Evidence shows that placental tissue is highly susceptible to oxidative stress caused by its extensive metabolic activity and other environmental insults. The excessive oxidative stress in the placenta, if not balanced by the antioxidant system, will damage placental function, leading to pre-eclampsia, intrauterine growth retardation of the fetus, and abortion [26]. Therefore, antioxidants have been widely used to prevent and treat placental dysfunction in animal models and also in human subjects [20,27]. Melatonin is a naturally occurring antioxidant. Different from other antioxidants, melatonin is an amphiphilic molecule being soluble in both water and fat [27,28]. This feature of melatonin makes it permeable to the placental barrier as well as to the embryo, with ease to act on both of them. This is an important reason that melatonin was selected in the current study to test whether melatonin supplementation to sows at their late stage of gestation would provide beneficial effects on the placenta via its antioxidant activity. Melatonin has been used to improve reproductive activity in different animals. For example, in sheep, the subcutaneous embedding of melatonin promoted the growth and development of lambs [29]. As mentioned above, Peng et al., gave melatonin to early gestated sows and also tested their oxidative stress responsibility [22]. Their study seems to have some similarities to ours. However, the targeted problems, the methods used and the findings were considerably different. We believe that early antioxidant intervention for pregnant animals like Peng et al., may do more harm than good by impairing angiogenesis and reducing the vascular density of the placenta. This is the reason that we gave melatonin to the pregnant sows at their late stage of gestation. We observed that this treatment potentially increased litter size and survival rate and significantly increased the birth weight as well as the weaning survival rate and weaning weight of piglets compared to the control. These beneficial effects of melatonin were not observed by Peng et al. Therefore, melatonin therapy should be used with caution in the early stages of normal pregnancy in pigs.

To explore the potential mechanisms, the effects of melatonin on the reproductive hormones, including progesterone and estrogen, are analyzed. Progesterone and estrogen are important hormones that regulate reproductive activities and establish and maintain pregnancy [30]. In this study, it is found that melatonin supplementation does not modify levels of blood progesterone and estrogen. The results are similar to that reported by Peng et al., and Lv et al. [22,31]. In addition, energy metabolism and cortisol level are also important factors for the reproductive performance of animals [32,33]. However, melatonin supplementation at the late gestational stage of sows also does not influence glucose and lipid metabolism, as well as the blood cortisol content, compared to the control. In this study, melatonin supplementation can significantly promote prolactin levels in peripheral blood during the prenatal period (112 days of gestation), which may improve the lactation performance of sows after delivery, but does not affect prolactin levels at 100 days of gestation. Similar to the properties of melatonin, prolactin synthesis also has an obvious circadian rhythm, suggesting that there may be a regulatory relationship between melatonin and prolactin. Misztal et al., showed that melatonin injection for 30 min resulted in increased prolactin levels in the peripheral blood of sheep [34]. Melatonin may be involved in the synthesis of prolactin, but the specific molecular mechanism remains to be further explored. Based on the above observations, the focus, then, is given to the effects of melatonin on the placenta. 

As mentioned previously, the placenta is the key structure to maintaining the fetus’s health, and it is also an important organ for melatonin synthesis during pregnancy [35]. The expressions of both melatonin synthetic rate-limiting enzymes AANAT and ASMT have been identified in human placental tissue. Meanwhile, melatonin receptors MTNR1A and MTNR1B are found in cytotrophoblast cells, syncytiotrophoblast cells and endothelial cells in the placental villi of humans [36]. Whether the melatonergic system is also expressed in the porcine placenta remains unknown. Here, we first report that the melatonergic system, including the mRNAs of AANAT, MTNR1A and MTNR1B, has been identified in the porcine placenta, and they are mainly expressed in trophoblast cells. In the current study, it was observed that melatonin supplementation at the late gestation stage mainly upregulated the expression of MTNR1B but not MTNB1A. The results suggest that the effects of melatonin on the placenta may be likely mediated by MTNR1B. 

To further explore the molecular mechanisms, placental tissues are sequenced by RNA. KEGG analysis of differential genes showed that the fluid shear stress and atherosclerosis signaling pathways were significantly enriched in the melatonin-treated placenta. The fluid shear stress pathway in the placenta can regulate the expression of the placental growth factor, which is an important regulator of placental angiogenesis [37]. Meanwhile, fluid shear stress can activate TRPV6 to promote placental microvilli formation [38]. Most importantly, fluid shear stress plays an important role in regulating the body’s antioxidant capacity [39]. It stabilizes Nrf2 and upregulates the expression of a spectrum of downstream antioxidant genes [40]. This observation has not been reported previously. The expressions of several downstream antioxidant genes of Nrf2, including MGST1, GSTM3 and GSTA4 in placental tissue, were significantly upregulated with melatonin supplementation compared to the control group. This observation is in line with the report of Peng et al. They also found that maternal melatonin supplementation increased the expression of antioxidant-related genes SOD, GPx1 and NQO1 in the placenta. The regulation of the Nrf2 signaling pathway by melatonin has been well documented. Under heat stress, melatonin activates the Nrf2 signaling pathway to improve the antioxidant capacity of Sertoli cells and alleviates heat-induced damage [41]. During cryopreservation of ovarian tissue, melatonin stimulates Nrf2/HO1 signaling pathway and inhibits ovarian oxidative stress and apoptosis [42]. Melatonin also reduces cadmium-induced damage in supporting cells by activating the Nrf2 signaling pathway [43]. Although many studies have documented the regulation effect of melatonin on the Nrf2 signaling pathway, the specific molecular mechanism has not been clarified. In this study, we have found that the activation of the Nrf2 signaling pathway by melatonin is probably mediated by the fluid shear stress pathway, particularly in sow placental tissue, thus improving the antioxidant capacity of placental tissue. This conclusion is further supported by the significantly decreased levels of lipid peroxidation product MDA in the serum of melatonin-supplemented sows compared to the controls.

## 5. Conclusions

**Figure 7:**
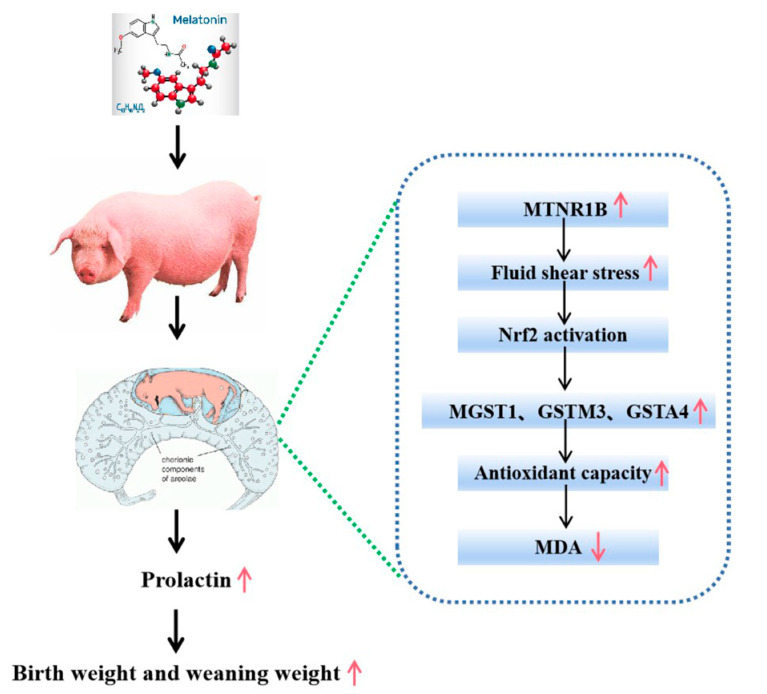
Patterns of increase in birth weight and weaning weight by melatonin supplementation in late gestation of sows (GD 90–GD 114).

In conclusion, melatonin supplementation during a late gestational stage in sows significantly improves the reproductive performance of the sows, including the potential increase in litter size, birth survival rate and significant increases in the birth weight as well as the weaning weight of piglets. These beneficial effects are mainly associated with the improved placental function of the sows with melatonin supplementation. Actually, the melatonergic system, including the mRNAs of AANAT, MTNR1A and MTNR1B, has been identified in the placenta of the sows. The potential molecular mechanisms involved in melatonin activate the placental tissue fluid shear stress pathway, which stimulates the Nrf2 signaling pathway and upregulates the downstream antioxidant genes to suppress the placental oxidative stress. Judging from the expression patterns of *MTNR1B* and *MTNB1A* with melatonin supplementation at the late gestation stage, that is that this treatment mainly upregulated the expression of *MTNR1B* but not *MTNB1A*. Therefore, we suggest that these effects may be mediated by the melatonin receptor of MTNR1B. This pathway is illustrated in (Figure 7).

## Data Availability

Data is contained within the article. The RNA-seq data reported in this paper are available in NCBI BioProject ID: PRJNA929422.

## Supplementary Materials

The following supporting information can be downloaded at: https://www.mdpi.com/article/10.3390/antiox12030688/s1, Table S1: The main primers of q-PCR.
