# Supplementary material for: Melatonin Supplementation during the Late Gestational Stage Enhances Reproductive Performance of Sows by Regulating Fluid Shear Stress and Improving Placental Antioxidant Capacity
Source: Antioxidants (Basel). 2023 Mar 10;12(3):688. doi: 10.3390/antiox12030688 (PMC10045541; doi:10.3390/antiox12030688)
Supplement: Supplementary file 1 [file antioxidants-12-00688-s001.zip › antioxidants-2262630-supplementary.pdf]

# Melatonin Supplementation during the Late Gestational Stage Enhances Reproductive Performance of Sows by Regulating Fluid Shear Stress and Improving Placental Antioxidant Capacity

**Likai Wang [1], Laiqing Yan [2], Qi Han [1], Guangdong Li [2], Hao Wu [2], Xiao Ma [2], Mengmeng Zhao [2], Wenkui Ma [2], Pengyun Ji [2], Ran Zhang [1] and Guoshi Liu [2,*]**

[1] State Key Laboratory for Agrobiotechnology, College of Biological Sciences, China Agricultural University, Beijing 100193, China
[2] National Engineering Laboratory for Animal Breeding, Key Laboratory of Animal Genetics and Breeding of the Ministry of Agricultural, Beijing Key Laboratory for Animal Genetic Improvement, College of Animal Science and Technology, China Agricultural University, Beijing 100193, China
* Correspondence: gshliu@cau.edu.cn; Tel.: +86-1891-1383-059

**Supplementary Table S1**

The main primers of q-PCR

| Gene | Sequences (Forward) | Sequences (Reverse) |
|---|---|---|
| MGST1 | GGACAGATGAAAGAGTGGAACG | GGGGAAGGGGTGTCAAATAC |
| GSTM3 | TTTCCTAACCTGCCCTATCTCA | TTTCTTCTTCAGTCTCCCCACA |
| GSTA4 | AGACGATCAGCAAAAGGAAGTG | TTGTCCATGACCCCGTAAAA |
| GSTA1 | ATGGTTGAGATTGACGGGATG | AATGCAGGAAGATAACGGTTTG |
| SOD2 | GTATCCGTCGGCGTCCAA | GCGGCGTATCGCTCAGTT |
| AANAT | CCCTGAGCCTGCCACCTAC | GGCGACCCTCCAAGAACC |
| MTNR1A | CCTGGTCATCCTGTCCGTGTA | TGAGACTGTGGCAGATGTAGCA |
| MTNR1B | CCCTGGTGCCCAACTTCTT | TTGTCCGACTTGACCTTCCTG |
